# Evidence of hard‐selective sweeps suggests independent adaptation to insecticides in Colorado potato beetle (Coleoptera: Chrysomelidae) populations

**DOI:** 10.1111/eva.13498

**Published:** 2022-10-25

**Authors:** Zachary P. Cohen, Yolanda H. Chen, Russell Groves, Sean D. Schoville

**Affiliations:** ^1^ Department of Entomology University of Wisconsin‐Madison Madison Wisconsin USA; ^2^ Department of Plant and Soil Sciences University of Vermont Burlington Vermont USA

**Keywords:** demography, insecticide resistance, pest evolution, positive selection, rapid evolution, recombination map

## Abstract

Pesticide resistance provides one of the best examples of rapid evolution to environmental change. The Colorado potato beetle (CPB) has a long and noteworthy history as a super‐pest due to its ability to repeatedly develop resistance to novel insecticides and rapidly expand its geographic and host plant range. Here, we investigate regional differences in demography, recombination, and selection using whole‐genome resequencing data from two highly resistant CPB populations in the United States (Hancock, Wisconsin and Long Island, New York). Demographic reconstruction corroborates historical records for a single pest origin during the colonization of the Midwestern and Eastern United States in the mid‐ to late‐19th century and suggests that the effective population size might be higher in Long Island, NY than Hancock, WI despite contemporary potato acreage of Wisconsin being far greater. Population‐based recombination maps show similar background recombination rates between these populations, as well as overlapping regions of low recombination that intersect with important metabolic detoxification genes. In both populations, we find compelling evidence for hard selective sweeps linked to insecticide resistance with multiple sweeps involving genes associated with xenobiotic metabolism, stress response, and defensive chemistry. Notably, only two candidate insecticide resistance genes are shared among both populations, but both appear to be independent hard selective sweep events. This suggests that repeated, rapid, and independent evolution of genes may underlie CPB's pest status among geographically distinct populations.

## 1 INTRODUCTION

The evolution of insecticide resistance is considered one of the most pressing issues facing insect pest management and sustainable agricultural practice. Despite considerable research effort over the last 40+ years (Georghiou & Taylor, 1977; Roush, 1989), evolutionary predictions from insect resistance management models have continued to underperform. In many cases, assumptions underlying these models (i.e., a single recessive resistance allele, fitness tradeoffs for resistance mutations, and a constant selection intensity from insecticides) might not apply to real‐world evolution of insecticide resistance given observations of large population sizes, high standing genetic diversity, and accumulation of resistance alleles over time (Bass et al., 2015; Brévault et al., 2013; Groeters & Tabashnik, 2000; Hoy, 1998). Consequently, pesticide resistance in herbivorous insects is still a major concern for the global food supply, as these pests cause an estimated 18%–20% damage (~$470 billion annually) to crops (Sharma et al., 2017). Research and development for new insecticides is becoming more difficult (Gould et al., 2018), and costs have more than doubled since 1995 (McDougall, 2018). However, knowledge of insect pest population genetics has been slow to develop, particularly in terms of understanding the genomic mechanisms and population genetic parameters that underlie resistance evolution (Pélissié et al., 2018).

Insecticide resistance phenotypes can be achieved through evolutionary changes in multiple pathways, such as: target‐site insensitivity (knockdown resistance), reduced penetration (cuticular change), xenobiotic detoxification and transport (metabolic processing by ABC transporters, cytochrome P450s, glutathione S‐transferases, and carboxylesterases), as well as behavioral avoidance of toxic compounds (Ffrench‐Constant, 2013; Mamidala et al., 2011; Scott et al., 1998). As a case in point, the Colorado potato beetle (CPB), *Leptinotarsa decemlineata*, shows evidence of at least two types of physiological resistance: target‐site insensitivity and metabolic detoxification (Alyokhin et al., 2008; Hawthorne, 2003). The evolution of target‐site insensitivity could be attributed to a single locus (Rinkevich et al., 2012), but metabolic detoxification seems to involve multiple molecular pathways (Clements et al., 2016; Zhu et al., 2016) and draws from different genes in those pathways across geographically dispersed resistant populations (Dively et al., 2020; Pélissié et al., 2022).

Colorado potato beetle is among the most notorious pests, with documented resistance to 56 insecticides (Whalon & Mota‐Sanchez, 2016) spanning all modes of action (IRAC, 2016), representing nearly 150 independent reports, and thus is an important model of agricultural pest genomics (Schoville et al., 2017). From early insecticides such as Paris Green and DDT, to modern insecticides such as pyrethroids and neonicotinoids, CPB populations have accumulated resistance over impressively short periods of time, even within 1 year (~2–3 generations) of the introduction of novel chemicals (Alyokhin et al., 2008; Brevik et al., 2018; Forgash, 1985; Ioannidis et al., 1991). In a species‐level comparative genomic analysis, CPB shows a higher rate of positive selection on putative insecticide‐resistant loci compared to other species in the genus *Leptinotarsa* (Cohen et al., 2020). Recent population genomic sequencing studies of CPB have found evidence for selection acting on many insecticide resistance pathways, supporting a prominent role of polygenic adaptation to insecticides over time (Crossley et al., 2017; Pélissié et al., 2022). Positive selection appears to act repeatedly on the same molecular pathways (and sometimes the same genes) across geographically isolated populations (Pélissié et al., 2022), but the signature of selection appears to be soft sweeps of polygenic traits on multiple haplotype backgrounds. Hence, these recent studies of CPB provide limited support for the classical insecticide resistance model that involves a single de novo mutation. Instead, results suggest that repeated pesticide management failures arise from high standing genetic diversity and polygenic genomic architecture of resistance traits, which can decrease latency while increasing likelihood of adaptation (Yeaman, 2022). If, in fact, field‐evolved resistance to insecticides occurs independently among geographically isolated CPB populations and arises from distinct genetic variation, employing general control strategies based on mutations in key target site genes will not lead to effective and sustainable control strategies at the population level.

These previous research efforts were based primarily on genome scans that leverage population differences in allele frequency to identify signatures of selection (Chen et al., 2010), but had limited power to detect hard selective sweeps due to the quality of genomic datasets, for example, contiguity, N50, number of scaffolds, etc. Recently, Cohen et al. (2021) generated a near‐chromosomal assembly of CPB, enabling improved analysis of linked variation along the genome. These advances motivated this investigation of selective sweeps and provided better inference of the demographic history and genome‐wide recombination patterns that contribute to genetic diversity and may confound signals of selection (O'Reilly et al., 2008).

The CPB's native range extends from southern Mexico throughout the plains and high desert regions of the United States (Jacques, 1988). Considered to be polyphagous, the CPB can survive on numerous Solanaceous plants, but its primary natal host is buffalo burr, *Solanum rostratum*. Potato beetle larvae and adults both defoliate plants and are therefore equally targeted by insecticides. The larval stage consumes nearly four times more leaf tissue than the adult stage (Ferro et al., 1985; Pelletier et al., 2011). Despite this expansive native distribution, it was not until the mid‐19th century that CPB expanded onto cultivated potato, *S. tuberosum*, whereupon it quickly spread eastward. The outbreak onto potato also introduced CPB to other cultivated Solanaceae, such as tomato, eggplant, and tobacco, where it is a minor pest (Jacques, 1988). During this rapid expansion, CPB quickly overwhelmed crops and became the target of early chemical control efforts and has remained a key species driving insecticide development and management practices (Alyokhin et al., 2015). We therefore focused our analyses on populations from Hancock, WI and Long Island, NY, two well‐studied, highly resistant populations that are geographically distinct and represent different stages in the geographical expansion of pest populations in the United States. Wisconsin potato feeding CPB are some of the first pest populations that migrated (1867) into the state following a major outbreak that started in Omaha, Nebraska (1859) and had ravaged neighboring Iowa by 1862 (Tower, 1906). These populations were observed to migrate eastward along a potato growing corridor and ultimately reached the Atlantic seaboard, and Long Island, NY, by 1872. They then invaded continental Europe and eventually spread to Asia (Grapputo et al., 2005; Tower, 1906). The CPB populations of New York represent the eastern expansion limit in the United States from the mid‐19th century outbreak and are notoriously pestiferous. Historic eye‐witness accounts describe how this population was a major nuisance due to its incredible size. Beetles would spill into Long Island Sound, floating in large mats that would swarm onto the hulls of anchored ships covering the decks and even causing noxious odors for beachgoers when they were washed ashore dead (Tower, 1906). The outbreak stopped a train at New York Central Railroad due to the potential hazard of their carcasses slicking the tracks! More recently, the Long Island population demonstrates cross‐resistance to multiple insecticides and the most rapid rate of resistance evolution observed in any CPB population (Alyokhin et al., 2015; Dively et al., 2020). Therefore, estimating genomic diversity and reconstructing the demographic history of these iteratively established pest‐populations is relevant to determine how colonization history and genomic diversity patterns influence contemporary resistance.

Levels of genome‐wide genetic variation are influenced by mutation, recombination, and selection, in direct relationship to effective population size (Hartl & Clark, 1997). Prior work suggests that CPB has relatively high genome‐wide standing genetic variation (Cohen et al., 2020; Pélissié et al., 2022; Schoville et al., 2017), yet it remains unclear how population history, recombination, and selection interact to shape this variation. Here, we examine the influence of demography and recombination on selection between two highly resistant geographically distinct pest CPB populations in the United States. We do this by estimating each populations' demographic history, generating population‐specific recombination maps, and testing for evidence of hard selective sweeps. We then compare the recombination hotspots to hard‐sweep regions within and between populations to assess the relative importance of novel mutations driving the evolution of insecticide resistance.

## 2 METHODS

### 2.1 Study sites and sampling

In order to determine how adaptation to insecticides has evolved in the CPB, we compared selection and recombination for two CPB populations known to be highly resistant to the neonicotinoid insecticide imidacloprid. Field‐collected beetles representing distinct geographical regions from: Hancock, Wisconsin (HAN: N 44.119753, W ‐89.535683) and Long Island, New York (LI: N 40.905657, W ‐72.752664) were collected at single sites, in the same growing season of 2015 (Table S1). Populations near Hancock Agricultural Research Station have been studied primarily due to their relatively high resistance to neonicotinoid (Imidacloprid®) insecticides (Clements et al., 2016; Crossley et al., 2017; Huseth et al., 2014), but they are also known to be resistant to several insecticidal chemistries (Dively et al., 2020). The LI population is historically extremely abundant and resistant to many insecticides, especially neonicotinoids, with nearly 30x fold resistance compared to susceptible samples (Alyokhin et al., 2015; Dively et al., 2020; Olson et al., 2000).

In this study, we generated novel whole‐genome resequencing data comprising 43 CPB adults (NCBI PRJNA753140), while combining it with previously published data from 10 adults (Accessions: SRR10388315 ‐ SRR10388319, SRR10388359 ‐ SRR10388363), for a total of 53 beetles (N_HAN_ = 28, N_LI_ = 25). For all samples, high‐molecular weight genomic DNA was isolated from thoracic muscle tissue using DNeasy Blood & Tissue kits (Qiagen).

### 2.2 Whole‐genome sequencing and variant calling

Whole‐genome sequence reads were generated at the University of Wisconsin Biotechnology Center, using either the Illumina HiSeq 2500 to generate 2 × 125 bp reads (previously published samples) or the Illumina NovaSeq 6000 platform to generate 2 × 150 bp reads (newly incorporated samples). Sequencing libraries were prepared using TruSeq DNA kits (Illumina). Sequencing effort was designed to yield >5x average coverage for each sample, a quantity sufficient to identify SNPs with reasonable accuracy (Lou et al., 2021; Nielsen et al., 2011). Samples were demultiplexed, adapters removed, and reads trimmed to remove low‐quality base calls using BBMap v38.7 (https://sourceforge.net/projects/bbmap/). Paired‐end read data were mapped to an updated reference genome from a nonpest individual sampled from Holly, Colorado (NCBI PRJNA750038) using BWA‐mem v0.7.17‐r1188 (Li, 2013; Li et al., 2009). Identification of polymorphic sites was carried out using ANGSD (Korneliussen et al., 2014), while applying filters for quality (at least 3x coverage per individual, with minimum and maximum total coverage across all individuals of 75x and 250x, respectively), base quality (−minMapQ 30), minor allele frequency (>0.05), and genotype probability (*p*‐value less than 1 e‐6). Given that different sequencing platforms were used to generate our read data, we tested for biases in allele frequency, which could influence variant detection (De‐Kayne et al., 2021). We sub‐sampled the Wisconsin population vcf by platform, isolating 1 million loci by position, and tested for significant differences in alternate allele frequency using a two‐sided Fisher exact test with a hypergeometric distribution in Microsoft Excel v16.65, as demonstrated in Ballian et al., 2009. However, we note that we expect deviation in the two datasets simply due to the variance in estimating allele frequency with a small sample size (*N* = 5 samples from the Illumina HiSeq 2500 platform). If alleles are common (*p* = 0.5), the expected variance for the estimate of allele frequency with a sample of *N* = 5 is ~2.5% (Fung & Keenan, 2014).

### 2.3 Demographic reconstruction and split time analysis

In order to reconstruct demographic history and estimate divergence time between these populations, we used two demographic reconstruction approaches based on the site frequency spectrum (SFS). Stairway plot 2 (Liu & Fu, 2020) was used to estimate recent demographic history from each population using the folded SFS generated in ANGSD. The dataset was restricted to neutral, intergenic sequences with stringent quality thresholds for coverage and likelihood (Supplemental Methods). We assumed two generations per year from observations of pest CPB voltinism at these latitudes (Jolivet et al., 1988) and, due to the lack of a specific CPB or related beetle mutation rate, we employed a recently determined mutation rate of 2.1 e^−9^ from the non‐biting midge, *Chironomus riparius*, which falls within the range of many insect species (Oppold & Pfenninger, 2017). Coleoptera and Diptera are sister groups that diverged from one another ~380MYA (Thomas et al., 2020). The second demographic method employed was based on a joint or 2D site frequency spectrum in dadi v2.1 (Gutenkunst et al., 2009). We used the dadi_pipeline v3.1.6 with model optimization (Portik et al., 2017) to reconstruct the split time for HAN and LI populations. A combined 2‐dimensional site frequency spectrum, 2D‐SFS, was generated from intergenic regions using the population specific SFS and combined using the ANGSD/realSFS programs, with downsampling projection carried out using easySFS (https://github.com/isaacovercast/easySFS) (Supplemental Methods). We used dadi_pipeline to compare three probable models of demographic history: (1) a divergence model with no migration but allowing for differences in effective size relative to the ancestor at the split time (no_mig), (2) the same model but allowing for an additional episode of instantaneous size changes during the evolution of each descendant lineage (no_mig_size), and (3) the no_mig divergence model but allowing for symmetrical migration (sym_mig). The models were defined based on current knowledge of the beetles' dispersal ability, as ecological dispersal rates are on average less than ~500 m per year (Boiteau et al., 2003) and genetic data suggest that populations do not interbreed over large distances. Despite historical evidence to the contrary, we still tested if migration between these geographically distinct populations could be inferred by the SFS via the sym_mig model (Grapputo et al., 2005; Izzo et al., 2018). These models are informed by a historical record of observations made by farmers and state entomologists that place time constraints on CPB's eastward expansion (1850 s‐1900 s, see Introduction; Tower, 1906). These models were subsequently tested with four consecutive rounds of optimization. Multiple replicates and parameter settings were used at each round with the default settings of dadi_pipeline (replicates = 10, 20, 30, 40; maxiter = 3, 5, 10, 15; fold = 3, 2, 2, 1), and parameters were optimized using the Nelder–Mead method (optimize_log_fmin). Replicate results were summarized using the Summarize_Outputs python script from dadi_pipeline and model selection was based on the Akaike information criterion (AIC; Akaike, 1973). To confirm that sample size (*N* = 53) did not influence model selection, a corrected AIC (AICc) was calculated for each dadi model using parameters based on Hurvich and Tsai (1989). Ancestral population size, *N*_*a*_, and divergence between HAN and LI were calculated using the formula *θ* = *4Ν*_*a*_ *μL*, where theta (*θ*) is provided from dadi output, mu (*μ*) is the midge mutation rate of 2.1 e^−9^, and the length (*L*) in base pairs includes all intergenic sequence data (~840 Mb). After solving for *N*_*a*,_ this value is multiplied by divergence time (*T*) and divided by the average number of CPB generations per year (2) to get approximate divergence in years. These parameters were averaged among the five replicates per model.

### 2.4 Estimation and comparison of population recombination maps

We generated fine‐scale recombination maps per scaffold per population in order to compare rates between populations and correlate selectively swept regions with contiguous haplotypes, that is, low recombining regions via pyrho v0.1.6 (Spence & Song, 2019). This program uses a composite‐likelihood approach to infer recombination maps from individual polymorphism data. The genotype data are used to compute a lookup table of two‐locus likelihoods of linkage disequilibrium, which are used to bind the hyperparameters of the model. An innovative feature of pyrho is that the lookup table accounts for demographic change by using independent estimates of effective population size over time while computing the two‐locus likelihoods.

To determine how population demography might support the single origin hypothesis for pest CPB populations, we used demographic estimates for each population from stairway plot 2 (see above). Scaffold‐specific population vcfs were used to estimate recombination in pyrho using a window size of 80 SNPs, with a step value 15 SNPs, for both populations. These parameters were determined from the pyrho_hyperparam function, while other parameters were left in default settings. The output from pyrho is an estimate of the per generation recombination rate per base (r). To convert to population recombination rates, we used the formula ρ = 4N_e_ r for autosomal scaffolds, where N_e_ is the effective population size estimate for each population from dadi. For the X‐chromosome scaffold (CPB has an XO system, where males lack a Y chromosome), we assumed no sex bias in the contribution to N_e_ and multiplied the autosomal effective size (4N_e_) by ¾ (Wright, 1984), while we adjusted the recombination rate (r) by ⅔ as recombination on the X chromosome only occurs in females (Lohmueller et al., 2010). The mean values of “r” per population for the entire genome were compared for significant difference using a Student's *t*‐Test in R v4.1.2 (R Core Team, 2021). Extreme high‐ and low‐recombination rates (>10 fold) were determined across the genome, relative to the average recombination rate of each scaffold. Recombination was also compared between coding and noncoding regions.

### 2.5 Selective sweep identification

Hard selective sweeps, which are named for their quick rise in frequency in a population, homogenous haplotype, and implicit fitness advantage, were identified for each population using the program RAiSD (Alachiotis & Pavlidis, 2018), with default parameter settings. Specifically, this program examines several selective sweep signatures across genomic windows, with a step size of 1 SNP, by measuring (1) high‐ and low‐frequency‐derived alleles, (2) localized LD on each flank of candidate a sweep locus, and (3) low LD between these flanking regions. RAiSD incorporates these metrics and generates a composite μ statistic that measures sweep intensity to exclusively identify hard sweeps. The analysis was run for each population, and the resulting outlier windows (highest μ values 0.05%) were filtered using a conservative threshold (α = 0.0005). To assess the false‐positive rate for selective sweeps at this threshold, we used neutral simulations with and without migration generated in the program ms (Hudson, 2002). Neutral instantaneous population growth was modeled using parameters estimated for HAN and LI, including estimated effective size (N_NY_ ~ 40,000 & N_WI_ ~ 15,000), divergence time converted to theta time units, MAF filtering of 5%, recombination rate, and mutation rate. Symmetrical migration between these populations was also modeled for the first 100 generations after divergence (Supplemental Methods). The resulting ms output were then used in RAiSD to determine thresholds for significant SNP outliers at 5%, 0.5%, and 0.05% cutoffs per population (Supplemental Methods).

Significant hard sweep regions were then annotated for overlap with coding regions using the intersect function in bedtools v2.26.0 (Quinlan & Hall, 2010). Both SNPs and genes were compared for overlap between the two pest populations. To understand the biological role of candidate genes and if they occurred in shared pathways, we examined the functions of their gene ontology (GO) terms. We conducted a GO enrichment analysis using a Fisher exact test with a hypergeometric distribution, with sampling by nonreplacement to assess significance (*p* < 0.05). Significant GO terms were grouped for similarity using VennPainter v1.2.0 (Lin et al., 2016). Visualization of significant GO terms was done using the web interface Revigo (Supek et al., 2011) and the python program Cirgo (Kuznetsova et al., 2019).

Finally, the distribution of outlier SNPs within shared genic regions was quantified and the size of the window surrounding each outlier region was also measured to support the identification by RAiSD of hard selective sweeps. The window size was taken from the RAiSD results, which reports the start and stop positions encompassing each region flanking a putatively swept SNP. We grouped the results as either hard‐swept regions (based on μ) or nonswept regions. We used a nonparametric Wilcoxon signed‐rank test (*W*) to calculate differences in the size of hard and nonswept regions.

## 3 RESULTS

### 3.1 Demographic reconstruction and Split time analysis

**FIGURE 1:**
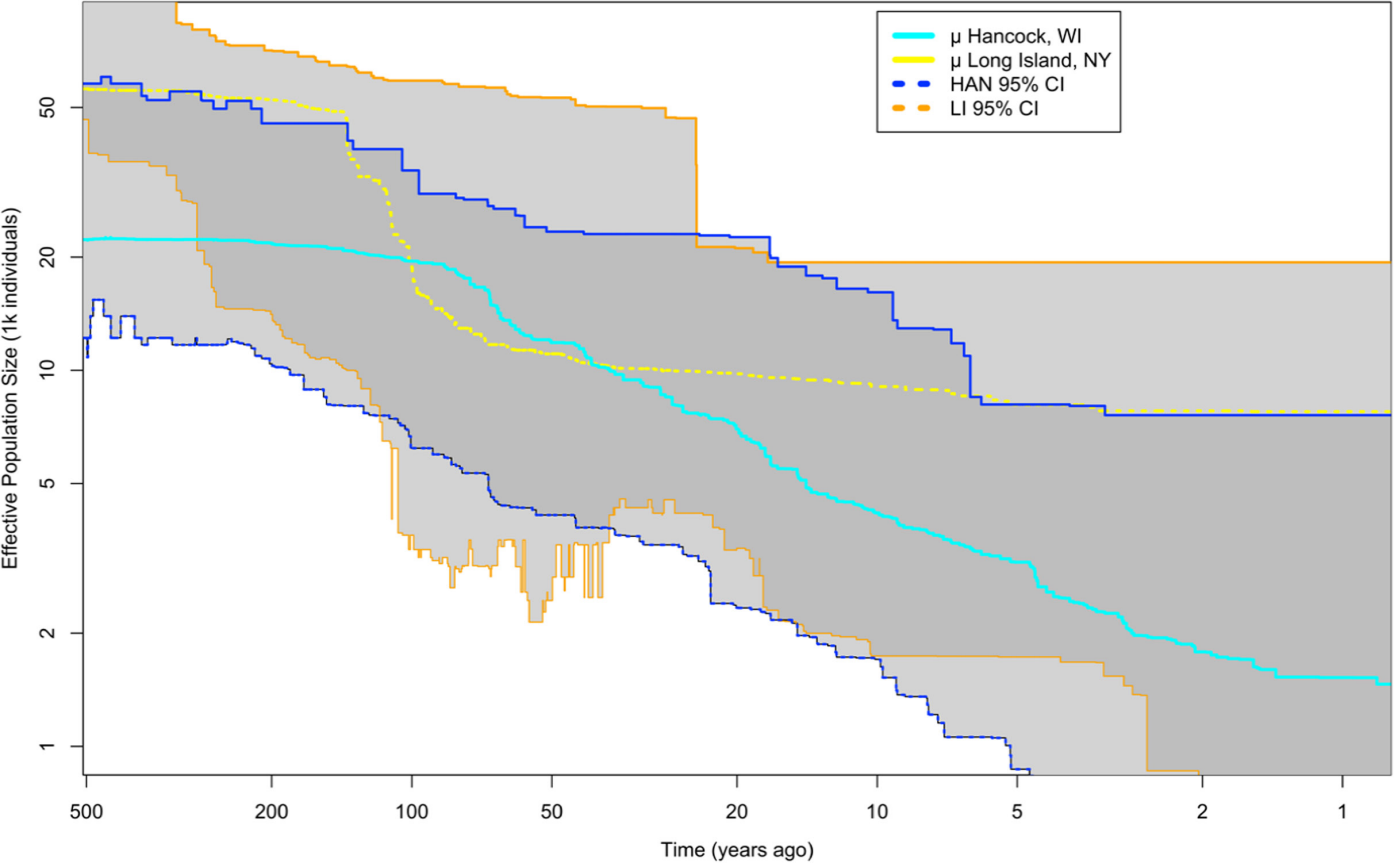
Demographic reconstruction of Colorado potato beetle population size change in Hancock, WI and Long Island, NY using stairway plot 2. Light shading represents the bounds of confidence intervals by population, darker shading is shared confidence intervals (CI) of both populations.

After cleaning the reads and curating polymorphisms for quality, the final call set retained 11.8 million and 12.4 million polymorphic sites for Hancock, WI and Long Island, NY populations, respectively of an approximately 870 MB genome (Table S1), with approximately 3% of SNPs having significant differences in allele frequencies between sequencing platform (Table S1). This is consistent with an expectation for sampling variance around allele frequency estimates due to the small number of samples (*N* = 5) sequenced on the HiSeq platform, so we conclude these data are unbiased and retain them in subsequent analyses. Stairway plot reconstruction shows that both the LI and HAN CPB populations have declined relative to their ancestral population sizes (Figure 1). The stairway plot estimated that the founding population for LI was about 10 times larger than the HAN. HAN (~90%) has declined proportionally more than LI (~85%), albeit with broad confidence intervals that overlap the range of effective size changes evident in LI. For the LI population, we estimated that the ancestral effective population size plateaued around 150 years ago, whereas the HAN plateaued more recently approximately 90–110 years ago (Figure 1). Using a model fitting approach to estimate the population split time, a no migration model with constant population size was preferred over a no migration model with variable size or a split with symmetric migration (AIC_no mig_ 86,303.16; AIC_c_ 86,303.56 < AIC_no mig size_ 93,710.02; AIC_c_ 93,710.43 < AIC_sym_mig_ 228,372.3; AIC_c_ 228,372.7), with the top five optimized no_mig replicates (Table S2). The constant size model was still favored after AICc. Parameter estimation suggested a divergence time of 159 ± 2.65 years ago and effective population size estimates of N_eLI_ = 40,071 ± 1447 and N_eHAN_ = 14,777 ± 572 individuals. This split model suggested a common ancestor with ~6700 individuals, with much larger contemporary populations in HAN and LI.

### 3.2 Estimating genome‐wide recombination rates for each population

**FIGURE 2:**
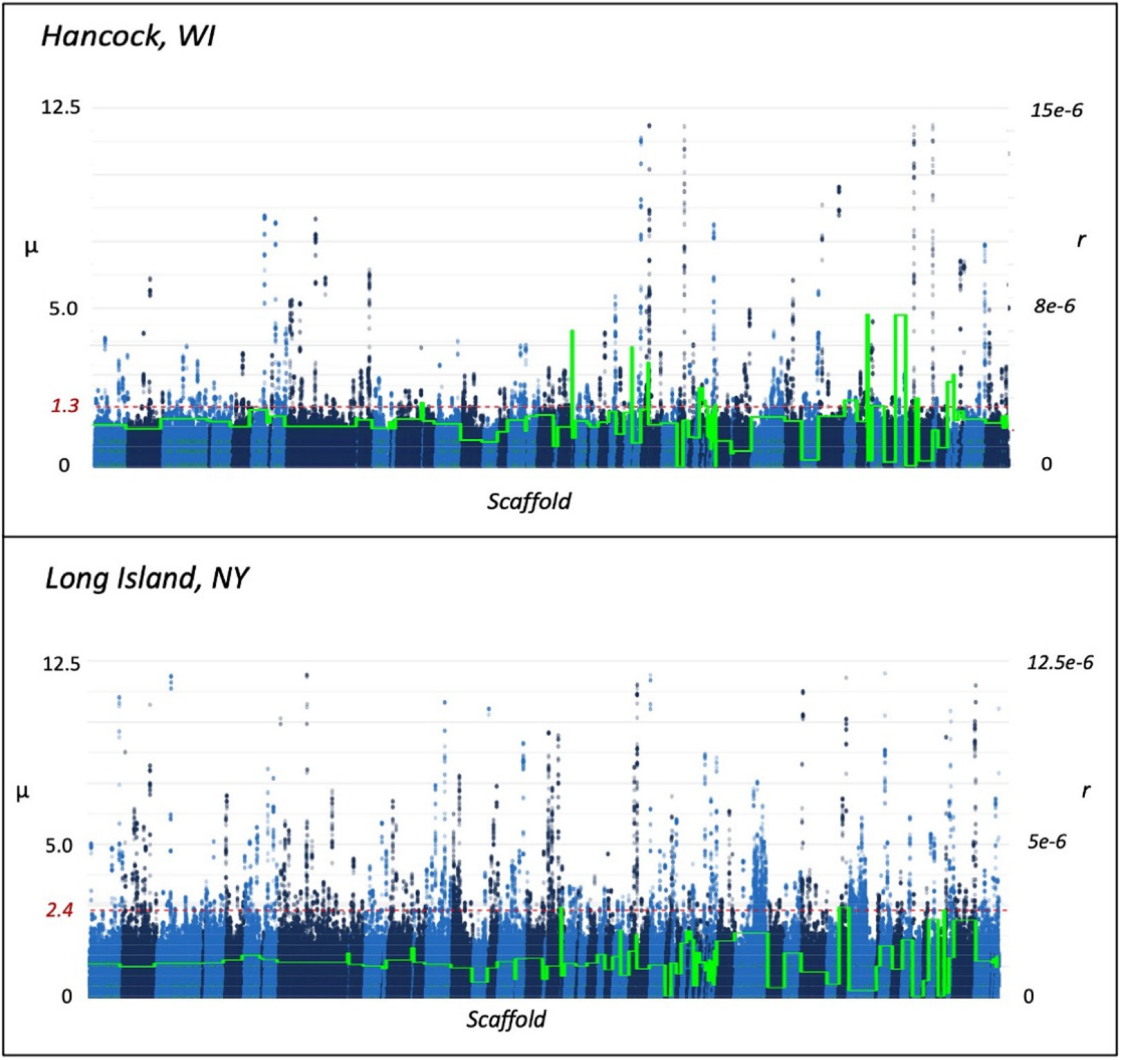
Scaffold averaged recombination of autosomal scaffolds, green trace (r), and selection (μ) with selection significance cutoff (0.05%) from RAiSD for Colorado potato beetle pest populations of Hancock, WI and Long Island, NY. The fine‐scale recombination map was estimated using a composite‐likelihood approach implemented in pyrho.

The population‐based recombination rate (ρ = 4N_e_ r) for HAN (ρ_HAN_ = 0.163) was significantly different at nearly one‐third of the LI (ρ_LI_ = 0.316) rate. The per‐generation per base recombination rate (r) differed only slightly yet was also significantly different (r_HAN_ = 2.75 e‐6 and r_LI_ = 1.95 e‐6; *p‐value <*2.2 e‐16). For the X chromosome, the population‐based recombination rate (for the X, ρ = 2N_e_r) was lower in HAN (ρ_HAN_ = 0.086) than LI (ρ_LI_ = 0.195), and much lower than the autosomal rates while the per generation per base rate (r) differed only slightly among populations in comparison to the autosomal rates (r_HAN_ = 2.91 e‐6 and r_LI_ = 2.4 e‐6). Recombination rates reflecting a 10‐fold difference above and below the average background scaffold recombination rate were compared among coding and non‐coding genomic regions. These rates were slightly higher in genic regions of HAN than nongenic regions (2.78 e‐6 > 2.69 e‐6), whereas rates in gene regions for LI were slightly less than nongenic regions (1.9503 e‐6 < 1.951 e‐6). The distribution among high‐ and low‐recombination regions (10‐fold difference from the mean) were compared between populations and examined for gene regions. There were fewer regions of high recombination (LI = 5295 and HAN = 5601) than low recombination (LI = 108,549 and HAN = 156,267, see Figure 2). A total of 447 genes occurred in shared regions of low recombination, while only three genes occurred in shared regions of high recombination (Table S3). Notable insecticide resistance genes in shared regions of low recombination include: several cytochrome P450s (CYP9e2, CYP6a23, CYP6a2, and CYPb‐c1), ABC multidrug resistance‐associated proteins, and nAChR subunit α1. One shared gene in a low‐recombination region (Niemann–Pick type protein, XP_023012615) has evidence of a shared selective sweep (see below) in both LI and HAN. Among the three genes in high‐recombining regions, one carboxylesterase gene (XP_023026553, LDEC012644) is potentially related to insecticide resistance.

### 3.3 Evidence of shared and unique population hard selected sweeps

**FIGURE 3:**
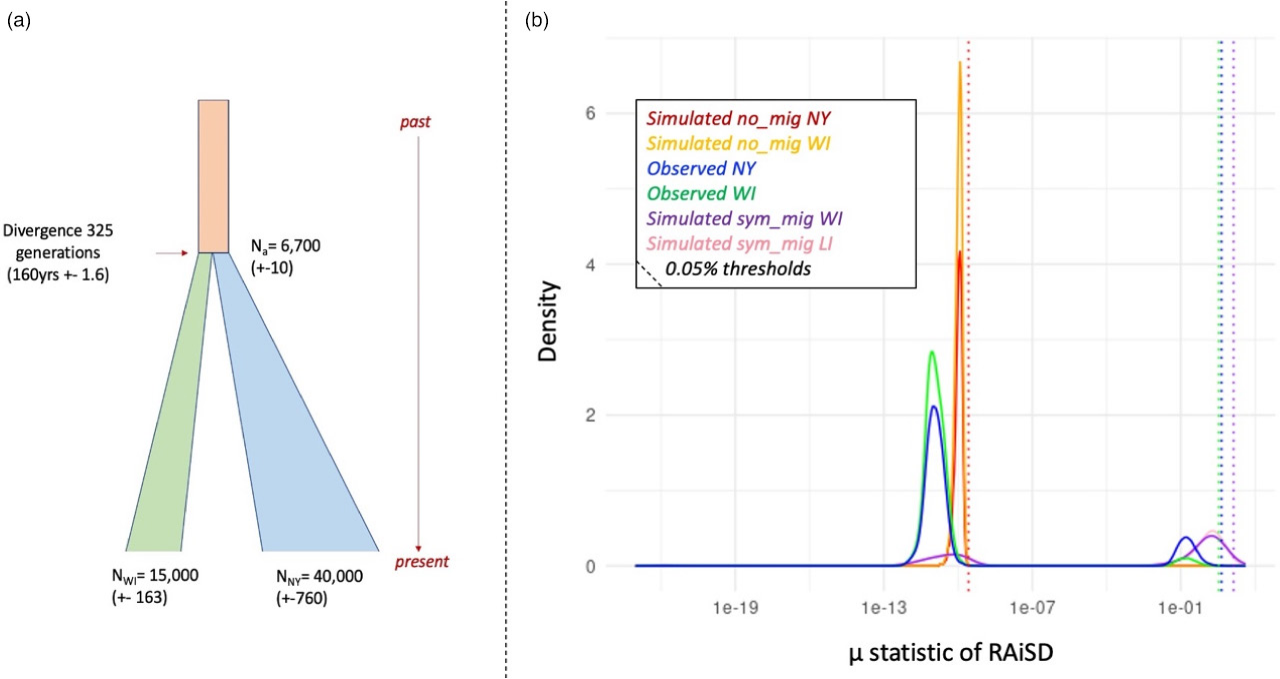
Evidence supporting hard selective sweeps. (a) Historical demographic model and inferred parameters used for neutral simulation in ms. (b) Simulated and observed selective sweeps from RAiSD (μ statistic, α = 0.0005) for Colorado potato beetle in Long Island, NY and Hancock, WI. The y‐axis indicates the probability density function for the kernel density estimation. The distribution of μ statistic values is shown on the x‐axis. Outlier values are indicated to the right of the x‐axis, where putative selective sweeps are defined as values exceeding cutoffs (dashed lines) for different sampling group under different observed or simulated conditions.

We examined both populations for evidence of hard selective sweeps using the μ statistic implemented in RAiSD, while assessing thresholds for the false‐positive rate from neutral simulations (Figure 3). A neutral no migration population growth model reconstructed in ms and tested in RAiSD generated a slightly broader distribution of μ than observed values, but there was no evidence of very large μ values, indicative of hard selective sweeps, unlike the observed data. The neutral model with moderate symmetrical migration, however, had a significantly skewed left distribution with a much higher μ, but again no clear second peak indicative of selection. Furthermore, the migration model was significantly unfavored as previously mentioned. When we defined a conservative 0.05% threshold based on the neutral simulations without migration (μ = 2.45 e‐10), we found approximately 450 k SNPs (~4% of the dataset) were significant in HAN and LI. Therefore, we opted for the much more conservative 0.05% threshold of observed data (μ = 1.3_WI_ & 2.4_NY_) for downstream analysis. There were more significant outlier SNPs (6211 with 1868 of these SNPs located within 89 genes) in the LI beetle population (Table S4) than in the HAN population that had 5932 SNPs with 1568 of these SNPs located within 74 genes. Population‐specific sweeps include agriculturally relevant genes that could contribute to pest success.

The LI and HAN populations shared only nine selectively swept genes, suggesting a minor role for convergent evolution or deeper‐time selection on shared ancestry (see Table 1), and two of these genes are isoforms of one another, residing within the same genomic region (E3 ubiquitin‐protein ligase HECW2 isoform X1 and X2). Among the 89 significant genes in LI, known insecticide resistance loci include: the neuronal acetylcholine receptor subunit alpha‐7 (XP_023013985, LDEC018602), cytochrome P450 9e2 (XP_023018113, isoforms X1, and X2), esterase FE4‐like (XP_023020747), and the alkaline phosphatase ALP‐1 (XP_023023329). Among the 74 significant genes in HAN, known insecticide resistance loci include: the segmentation protein cap‐n‐collar (XP_023016920), an ATP‐binding transporters (ABCD3: XP_023011673 and LDEC007110, ABCC: XP_023026978), salicyl alcohol oxidase (LDEC016943), and an esterase (LDEC022531). Among the nine genes within hard swept regions shared by both populations, there are two candidate insecticide resistance genes: an ABC transporter (ABCC: XP_023026978, LDEC019090) and a carboxylesterase (XP_023025254, LDEC022531).

**TABLE 1:** Shared candidate genes identified as hard selective sweeps in RAiSD in both Hancock, WI and Long Island, NY

NCBI gene	Gene annotation	Recombination rate ρ	SNPs	Length of swept region (bp)
XP_023014225	Paired mesoderm homeobox protein 2A‐like	0.227 (WI) 0.281 (LI)	40 (WI) 25 (LI)	24,702 (WI) 24,643 (LI)
XP_023018886	E3 ubiquitin‐protein ligase HECW2 isoform X1	0.159 (WI) 0.244 (LI)	26 (WI) 26 (LI)	11,011 (WI) 11,959 (LI)
XP_023018894	E3 ubiquitin‐protein ligase HECW2 isoform X2			
XP_023012615	Niemann–Pick type protein homolog 1B‐like	0.265 (WI) 0.082 (LI)	9 (WI) 2 (LI)	8551 (WI) 9711(LI)
XP_023020349	Uncharacterized protein LOC111508933	0.176 (WI) 0.089 (LI)	20 (WI) 21 (LI)	20,332(WI) 16,327(LI)
XP_023025254	Uncharacterized protein LOC111513291	0.181 (WI) 0.0.229 (LI)	47 (WI) 29 (LI)	42,930 (WI) 42,228 (LI)
XP_023025815	Neuroendocrine convertase 2‐like	0.183 (WI) 0.405 (LI)	1 (WI) 33 (LI)	34,166 (WI) 33,273 (LI)
XP_023026978	Multidrug resistance‐associated protein 1	0.194 (WI) 0.277 (LI)	51 (WI) 2 (LI)	61,771 (WI) 61,777 (LI)
XP_023027618	GMP synthase	0.076 (WI) 0.286 (LI)	33 (WI) 23 (LI)	42,468 (WI) 42,740 (LI)

Gene ontology enrichment resulted in 281 significant terms unique to LI and 169 significant terms unique to HAN, with 27 shared significant terms. While shared enriched GO terms only included a few relevant to insecticide resistance (include calcium ion binding GO:0005509 and transmembrane transport GO:0055085), terms within populations included: HAN: ATPase‐coupled transmembrane transporter activity GO:0042626, larval feeding behavior GO:0030536, cellular oxidant detoxification GO:0098869; and LI: ATP‐dependent peptidase activity GO:0004176, chloride channel activity GO:0005254, oxidation‐dependent protein catabolic process GO:0070407, and voltage‐gated cation channel activity GO:0022843 (Figures S1–S7; Table S5).

The distribution of nucleotide variation and length of sweep regions provides information about the pattern of selective sweeps in the genome. SNP abundance in each sweep region and the size of the region was quantified, with HAN having a higher average number of SNPs (23.4/swept locus) compared to LI (19.6/swept locus). The top 0.05% swept regions were significantly larger than non‐swept regions for both HAN (HAN swept_u_ = 37,512 >> nonswept_μ_ = 3524; *W =* 1.8e9, *p*‐value < 2.2 e‐16) and LI (LI swept_μ_ = 51,138 >> nonswept_μ_ = 3361; *W* = 2.2e7, *p*‐value < 2.2 e‐16) (Figures S8). However, the size of the shared sweep regions (that possess a shared significant outlier SNP within 1 bp for both HAN and LI) was not significantly larger than the average sweep regions (*W* = 84,475, *p*‐value = 0.366).

## 4 DISCUSSION

There is significant interest in understanding how demography and neutral genetic processes influence population diversity and adaptation (Hawkins et al., 2019; Kreiner et al., 2018; Pélissié et al., 2018). An archetype of this phenomenon is rapidly evolving insect pest populations, which can serve as a proxy for nonpest systems that may also experience rapid environmental change. To more accurately understand how adaptation in a population might have occurred, reliable genetic and demographic estimates of population size, mutation rate, and recombination rate are necessary. Therefore, we first estimate these parameters and test for evidence of hard selective sweeps in two rapidly adapting pest populations of the CPB.

Classical expectations for single locus adaptive mutations have been challenged by growing evidence of polygenic resistance traits (Kreiner et al., 2021; Pélissié et al., 2022; Wybouw et al., 2019). However, there are well documented examples of both single locus and polygenic resistance evolution (Ffrench‐Constant, 2013) and the relative importance of these selective regimes might relate to fundamental population genetic properties (Barton, 2010; Hermisson & Pennings, 2005). Here we provide evidence that hard selective sweeps occur in CPB at unique loci attributed to distinct resistance mechanisms and occur independently in geographically isolated populations. Identifying these independently derived hard sweeps contributes to our understanding of CPB adaptation, along with prior studies that suggest soft selective sweeps are drawn from standing variation across the range of CPB (Crossley et al., 2017; Pélissié et al., 2022). Additionally, through an analysis of demographic history and recombination patterns, we provide an improved understanding of pest population history and sources of genomic variation in CPB, which will improve predictive modeling of resistance evolution in agricultural ecosystems.

### 4.1 Demographic history of Colorado potato beetle Pest populations

In contrast to introduced pests, which experience a genetic bottleneck prior to invasion (North et al., 2021), native pests are thought to retain much of their genetic variation. Unlike other major agricultural pest species, CPB experienced a host expansion onto potato and switched to pest status in part of its endemic range, North America (eastern Nebraska) in 1859 (Walsh 1866). The host expansion of CPB allowed for the retention of high standing genetic diversity as the pest extended its native range eastward, as opposed to a reduction of genetic diversity associated with a founder event (Cohen et al., 2020; Izzo et al., 2018; Pélissié et al., 2022). Geographical structure was shown in earlier population genetic studies using microsatellites, AFLPs, and mitochondrial DNA (Grapputo et al., 2005; Izzo et al., 2018). Our models suggest that CPB pest populations in the Midwest and Eastern US diverged from a founder population of approximately ~6700 individuals nearly 160 years ago (~320 generations) and increased in size in Wisconsin (2.2‐fold) and New York (5.5‐fold). Contemporary work with whole‐genome resequencing data, albeit at much smaller population sample sizes, suggests large effective population sizes occur throughout the pest range of CPB (Pélissié et al., 2022).

Our conservative thresholds for SNP ascertainment and sample sizes are reasonable to reliably determine effective size per population, yet given the low sequencing depth per individual, we might expect possible heterozygous sites to be mistaken as homozygous (Fumagalli, 2013). This can influence the SFS by reducing singletons and causing a bias that results in underestimating demography. As the SFS is integral to estimates of demography and recombination (less so in RAiSD, given this metric relies on linkage disequilibrium and variance between swept regions, in addition to the SFS), our results might lead to an underestimate in effective population size (Crawford & Lazzaro, 2012). We based our analysis of recombination on the dadi estimates, which were ~ 4‐fold larger than estimates from the Stairway plot analysis. The dadi estimate was obtained on the full joint‐SFS under assumptions of a constant size demographic model. In our Stairway plot analysis, we ignored genic regions and recovered a declining population. While there is uncertainty about the true effective size, we note that assuming slightly larger effective size should affect population‐based recombination rate (ρ) and not the genome‐wide pattern of recombination.

The migration history, contemporary land usage and pest management practices differ significantly between Wisconsin and New York. Historically, potato acreage in Wisconsin was rather dispersed, but covered approximately 3 million acres (~1200 km^2^) in the early 20th century (Crossley et al., 2019). It subsequently became concentrated in several growing regions comprising ~75,000 acres (300 km^2^) today. This contrasts with the potato growing history of Long Island, which was very concentrated and encompassed 70,000 acres or ~300 km^2^. Present‐day estimates suggest potato farming comprises only ~300 acres (~1.25 km^2^; Alyokhin et al., 2015). While this dramatic reduction in potato growing acreage (habitat) might account for the steep decline in population size that was estimated to occur 50–100 years ago (Figure 1), as well as the slight reduction in the number of SNPs identified within swept regions, this timing is also coincident with the introduction of modern synthetic insecticides. The variability of the lower confidence interval for LI might be due to a lack of coalescent events that provide an estimate for effective population size. Resistance history to insecticides varies between HAN and LI, as the Long Island populations developed resistance to every major class of insecticides and have the highest tolerance to novel chemicals of any CPB population (Olson et al., 2000).

There has been some debate about the dynamics of this species' range expansion, particularly whether it involved admixture among multiple non‐pest source populations (Izzo et al., 2018), or a much earlier colonization of the Midwestern US than recorded in the literature (Pélissié et al., 2022). Our estimate of ~160 years of divergence between Wisconsin and New York populations approximates the historically observed expansion of CPB onto cultivated potato in the 1850s and within a small margin of error for the known CPB occurrence in both states (Izzo et al., 2018; Tower, 1906). During the mid‐ to late‐19th century, there was a large continuous band of cultivated potato extending from Nebraska to the Midwest and Eastern US (Crossley et al., 2020), which provided a dispersal corridor for the pest lineage to quickly spread to the Atlantic seaboard by 1874 (Casagrande, 1987; Riley, 1877). While the effective population size trajectory through time differs between LI and HAN, with LI having a larger effective population size (supported by both dadi and stairway plot results), there is broad overlap in confidence intervals and a convergence in estimates around the ancestral population size.

In our dadi analysis, a population size model that included an additional episode of instantaneous change after population divergence was not favored over the simpler split model. This split model yielded population size estimates several fold larger in the descendent populations (HAN and LI) than the shared ancestral population. However, the stairway plot suggested a decline in effective population size for both populations. The discrepancy between the two results is most likely explained by the differences in the demographic reconstruction algorithm, where stairway plot calculates a composite likelihood for population size change over a flexible multi‐epoch model and dadi uses a diffusion approximation within a fixed demographic model. While the estimates for demographic history vary, the stairway plot estimates result in large confidence intervals around the effective size mean and this average does encompass our estimates from dadi (Mazet et al., 2016). Other factors, such as gene flow from a structured population, could generate a spurious signature of a recent bottleneck. In a previous study, Crossley et al. (2017) showed that Wisconsin populations were weakly structured. However, it is not clear that this accounts for the declining population size trajectory of CPB samples from Hancock. Perhaps ecological factors, such as a declining extent of Wisconsin potato acreage in the late 19th century (Crossley et al., 2020), may explain reductions in effective size since colonization.

### 4.2 Regional differences in recombination

We found similar levels of the per‐generation recombination rate (r) in both HAN and LI, although the population‐based recombination rate (ρ) is higher in LI due to its larger effective population size estimate. Both populations have regions of high and low recombination, with many more low‐recombining regions. Low‐recombining regions may be indicative of positive or purifying selection, and these regions intersect genes associated with different modes of insecticide resistance, including target site insensitivity (glutamate receptors XP_023012213 and XP_023012139, which are targets of the abamectin IRAC MOA Group 6) and detoxification (carboxylesterase XP_023018836 and CYP genes including one CYPb‐c1, two CYP6a2, three CYP6a23, and five CYP9e2s). CYPs have been repeatedly associated with metabolic detoxification of xenobiotics (Bass & Field, 2011; Ffrench‐Constant et al., 2004; Scott & Wen, 2001), resistance to particular insecticidal modes of action (Amichot et al., 2004; Daborn et al., 2001), and cross resistance to multiple modes of action (Peng et al., 2016; Zhu et al., 2016).

Our estimates of population‐based recombination rates (ρ = 4Ne r) ranged from 0.163 to 0.316, while rates for the X chromosome (ρ = 2Ner) ranged from 0.086 to 0.195. To our knowledge, these are the first recombination rate estimates based on population genomic data for Coleoptera. The rates are similar to estimates from Anopheles (Nelson et al., 2021), but approximately an order of magnitude lower than Drosophila melanogaster (Chan et al., 2012) and several bee species (Jones et al., 2019). However, it is well known that recombination rates can vary widely among closely related species, populations, and even between the sexes (Kong et al., 2010; Smukowski & Noor, 2011; Stapley et al., 2017).

### 4.3 Hard selective sweeps and models of insecticide adaptation

Metabolic detoxification has emerged as the most ubiquitous type of resistance in arthropod pests, which may not be surprising given its diversity of molecular pathways and enzyme families, as well as its evolutionary function in protecting arthropods against plant allelochemicals (Li et al., 2007). Detoxifying enzymes are generally classified into three phases based on their relationship to xenobiotic substrates (Heckel et al., 2007; Li et al., 2007). Phase I: functionalization mediated by cytochrome P450s (CYPs); Phase II: conjugation to hydrophilic substrates mediated by glutathione‐S‐transferases (GSTs) and esterases; and Phase III: excretion mediated by ATP‐binding cassettes (ABC transporters). Target site insensitivity, usually involving a point mutation, is another prevalent mechanism of xenobiotic resistance that has been associated with pyrethroid and DDT resistance in *Musca domestica* (Rinkevich et al., 2006, 2012), *Anopheles gambiae* (Ranson et al., 2000) *Culex pipiens* (Martinez‐Torres et al., 1999), *L. decemlineata* (Hawthorne, 2001), and tobacco budworm, *Heliothis virescens* (Park & Taylor, 1997).

Using parameter estimates to generate neutral data under a population growth model, with and without migration for each population, we ascertained probable false‐positive rates for the μ statistic in RAiSD at cutoffs of 5%, 0.5%, and 0.05%. A growth model without migration was chosen to be conservative, as our demographic reconstruction from dadi favored such a model (Table S2). No outliers of selection (large μ values) are identified in the neutral no‐migration simulations (Figure 3). In contrast, simulated migration between the populations for ~100 generations increased the false‐positive rate (but generated a broad distribution of μ values), which is expected as introgressed haplotypes appear as rare alleles with extensive linkage disequilibrium (Figure 3; Supplemental Results). As migration was not favored by SFS reconstruction, we opted to use a 0.05% threshold based on the no‐migration simulation for our observed SNP data sets to identify the most strongly supported hard sweeps. Our observed data show a distinct peak of large μ values for both LI and HAN. This distinct bimodal pattern (Figure 3 observed data) is different from both simulated neutral models, suggesting selective sweeps leave a distinct signature in our data. Both pest populations showed signatures of strong selective sweeps at genes associated with agricultural adaptation. While the genes involved in the selective sweeps were unique to each population, the genes had similar roles in detoxification, host detection, and defense, based on GO (Tables S3‐S4). Of the nine shared genes, two have an association with insecticide resistance: a carboxylesterase (Phase II; XP_023025254) and an ABC transporter (Phase III; XP_023026978), while the other shared genes are associated with neuronal development and function, GMP synthase and metabolism (Table 1). We acknowledge that given the shared demography of these populations, the signatures of selection at these alleles could predate historical insecticide use, so further work is needed to clarify the age of selected loci.

Genes in these protein families have been linked with pesticide detoxification in CPB and other insect pests (Bhatt et al., 2020; Cohen et al., 2020; Crossley et al., 2019; Jao & Casida, 1974; Lü et al., 2015). The Long Island population is notoriously resistant to insecticides (Alyokhin et al., 2015; Dively et al., 2020). Tolerance to novel chemistry has been attributed to constitutive overexpression of detoxification genes (CYPs, GSTs, and esterases, among others; Clements et al., 2018). However, one of the unique swept loci in the LI population is a major target site for neonicotinoids, the nicotinic acetylcholine receptor subunit. However, this is not differentially expressed in resistant samples (Dively et al., 2020), despite this population having much higher resistance for imidacloprid than HAN in Wisconsin. A CYP locus (CYP9e2) was also swept in the LI population and is among other CYPs in shared low‐recombination regions with HAN. For HAN, sweeps include a known trans‐regulatory element for imidacloprid resistance (cap ‘n’ collar), which induces co‐expression of ABC transporters, GSTs, CYPs, and esterases (Gaddelapati et al., 2018). The Hancock population also has evidence of selection for a biologically integral defensive enzyme (salicyl alcohol oxidase, LDEC016943), which catalyzes the formation of a volatile deterrent salicylaldehyde in *Chrysomela tremulae* and *C. populi* larvae (Michalski et al., 2008). Its role and relevance in CPB has not been determined.

Relative to other studies that have identified selective sweeps in insect pests, we identify more hard swept regions, yet a similar number of genes within these swept regions (Calla et al., 2021; Nam et al., 2020; Weedall et al., 2020). Until recently, most research on CPB focused on well‐known target genes and metabolic pathways (Alyokhin et al., 2008). As next generation sequencing technology and genomic resources have become available for CPB (Cohen et al., 2020; Kumar et al., 2014; Schoville et al., 2017), there has been a rapid expansion of knowledge into the genetic basis of pesticide resistance using genome scans as an agnostic approach to gene discovery. With respect to prior comparative population genetic studies of CPB, which had presented extensive candidate gene lists (>8700 genes from a more inclusive demographic sampling; Pélissié et al., 2022), this work suggests a small set of genes (<150 per population) undergo hard selective sweeps. Compared to Pélissié et al. (2022), we find that ABC transporters and an esterase, swept in both NY and WI, were also significant in their genome scan results. Additionally, 13 and 17 genes from their PCAdapt analysis are shared in our analysis of HAN and LI samples, respectively (Table S4). The size of the swept regions around significant SNPs is larger than the average size of all regions around all SNPs. The distribution and number of SNPs between these populations suggests that they are distinct haplotypes that have been selected independently in each region. Together, these results imply a combined effect of newly determined hard and previously described soft selected sweeps on CPB genes that support agricultural adaptation maintained by large population size and standing genetic diversity.

### 4.4 The legacy of demography with recombination and selection

Genomic regions with little genetic diversity, such as fixed haplotypes, might be a result of a demographic bottleneck or inbreeding event, migration, purifying or positive selection, or a lack of recombination (Jensen et al., 2005). Selection patterns are typically interpreted considering demography, as recombination fine‐mapping approaches have only recently become feasible for nonmodel species (Spence & Song, 2019). Here, we sampled two divergent and geographically isolated pest populations that have persisted in similar agroecosystems to identify genomic regions under selection. By first demonstrating that divergence between our sample populations is consistent with historical observations, we were then able to find selected sites that have arisen independently within these populations. The candidate genes were selected for after population divergence, as they reside in population specific loci with few polymorphisms. However, only a few (7/89 in HAN and 3/132 in LI) of these loci coincide with our significantly (>10‐fold) shared low‐recombination regions suggesting that the detection of these loci is not confounded by low‐recombination rates. Interestingly, the difference in effective population size for these groups does not readily explain phenotypic patterns observed today. Although the HAN population has a lower recombination rate, smaller N_e_, and subsequently fewer genes undergoing selection than LI, it is not more susceptible to insecticides. Both populations are effectively controlled by novel chemistries in the field despite significant in vivo LC_50_ differences (Dively et al., 2020). While measures of genetic diversity and effective population size are reliable proxies for adaptive potential, they are strongly influenced by demographic history. When it comes to pest control, these parameters could drive improved decision‐making, with the goal of effective, sustainable, and population specific control methods.

### 4.5 Leveraging population genomics for integrated Pest management

The ability of CPB to rapidly evolve resistance continues to challenge the sustainability of potato production (Alyokhin et al., 2015), which is an economically important vegetable crop with an annual production valued at $3.91 billion (USDA NASS,  2022). Due to a general lack of effective natural enemies of CPB, potato production has historically depended upon insecticides to control the beetle (Hare 1990). Without a clear understanding of *how* CPB and other successful pests continue to evolve resistance, resistance will continue to develop in a matter of generations and growers will lack effective strategies to manage CPB over the long‐term. Interestingly, we determine similar selective regimes in divergent populations despite different management practices and measured insecticide tolerances. Expanding baseline knowledge of the genetic loci and gene networks involved in resistance can help mitigate the spread of resistance into susceptible populations by allowing for biomonitoring. However, a potentially more important outcome of this work is to enable a refinement of IPM strategies for the future use of novel pesticidal chemistries. Improved understanding of population genetic parameters, including their variation among geographical regions, allows for more effective predictive modeling of resistance evolution (Karlsson Green et al., 2020). Drawing on examples of insecticide, fungicide, and antibiotic resistance, Beckie et al. (2021) have argued that efforts to minimize selection and impede dispersal in taxa that exhibit cross‐resistance may need to be tailored to specific pest species for improved IPM outcomes. Due to substantial standing genetic variation and a tendency to locally evolve resistance by both hard and soft sweeps, management of CPB should leverage modeling approaches that examine not only single large effect de novo mutations, but also accumulation of polygenic resistance traits from background genetic diversity (Haridas & Tenhumberg, 2018) in discrete regional pest populations.

## CONFLICT OF INTEREST

The authors declare no conflict of interests.

## Supporting information


**Appendix S1:** Supporting Information

## Data Availability

Raw genomic read data for each population have been deposited on NCBI (PRJNA753140).
